# Correlation between remnant cholesterol and hyperuricemia in American adults

**DOI:** 10.1186/s12944-024-02167-0

**Published:** 2024-06-08

**Authors:** Xiaohai Zhou, Xiaolu Weng, Jing Xu, Wenxing Wang

**Affiliations:** 1 Department of Hematology, The Second Affiliated Hospital and Yuying Children’s Hospital of Wenzhou Medical University, Wenzhou, Zhejiang Province P. R. China; 2 Department of Endocrinology, The Second Affiliated Hospital and Yuying Children’s Hospital of Wenzhou Medical University, Wenzhou, Zhejiang Province P. R. China; 3 Department of Gastroenterology, The Second Affiliated Hospital and Yuying Children’s Hospital of Wenzhou Medical University, Wenzhou, Zhejiang Province P. R. China

**Keywords:** Remnant cholesterol, American, NHANES, Hyperuricemia, Uric acid

## Abstract

**Background:**

Remnant cholesterol (RC) is an important marker for assessing the risk of metabolic syndrome. However, the correlation between RC and hyperuricemia (HUA) remains unclear. This study aimed to explore the correlation between RC and HUA in American adults.

**Methods:**

A total of 9089 participants from the 2013–2020 National Health and Nutrition Examination Survey were investigated. The correlation between RC and the odds of HUA was evaluated using multivariate logistic regression analysis. The nonlinear correlation was described using fitted smoothed curves. The correlation in subgroups was analyzed based on race, gender, alcohol consumption, age, body mass index, waist circumference, diabetes and moderate physical activities.

**Results:**

RC was correlated with uric acid (Spearman’s correlation coefficient = 0.208 in males and 0.215 in females; all *P* < 0.001). Multiple logistic regression analysis indicated a positive correlation between RC and the risk of HUA (odds ratio = 1.022 in males and 1.031 in females; all *P* < 0.001). Subgroup analysis revealed that the correlation was stronger in females, participants aged < 50 years, and those without diabetes. Furthermore, the generalized smooth curve fitting demonstrated a linear correlation between RC and HUA, without threshold or saturation effects.

**Conclusion:**

Elevated RC significantly and positively correlated with HUA in American adults. This correlation was stronger among females, participants aged < 50 years, and those without diabetes.

## Introduction

Hyperuricemia (HUA), characterized by the excessive production or inadequate excretion of uric acid, is a significant global health concern closely associated with gout and a variety of other medical conditions, impacting individuals of all genders and ages [1, 2]. HUA can serve as an independent risk factor for numerous systemic diseases, such as cardiovascular diseases, gout, chronic kidney disease and hypertension [3]. As of 2016, the prevalence of HUA has reached 21% worldwide [4], ranging from 14.6 to 20% in the United States [5]. Notably, there is a trend affecting younger individuals, with mean age of 38.6 years old. HUA can impose substantial health burdens on public health infrastructure [6]. Hence, it is imperative to develop a straightforward and expeditious detection technique for timely identifying individuals at heightened risk of HUA, enabling targeted interventions for disease prevention.

RC, an innovative atherogenic lipoprotein, refers to the cholesterol content present in triglyceride-rich lipoproteins, predominantly comprising chylomicron remnants, quite low-density lipoproteins, and intermediate-density lipoproteins. Typically, RC can be determined by subtracting the levels of high-density lipoprotein cholesterol (HDL-C) and low-density lipoprotein cholesterol (LDL-C) from Total cholesterol (TC), as calculated from a standard lipid profile [7]. Notably, mechanistic evidence indicated that elevated concentrations of RC are associated with low-grade inflammation, which are genetically affected by insulin resistance (IR) [8–10]. A study of a subject on the epidemiology demonstrated that as the level of RC increases, there will be a corresponding increase in the prevalence of T2DM, hypertension, and lipid disorders [11–14]. Furthermore, the correlation between RC and MetS is characterized by a positive feedback loop involving IR, chronic inflammation, hypertension and abnormal lipid metabolism. RC has the impact on these factors and its reciprocal correlation with the results in the accelerated progression of MetS [15–18].

Previous studies have explored the correlation between conventional lipid parameters such as TC or triglycerides (TG) and HUA [19–22], while the precise correlation between RC and HUA remains unclear. Consequently, this study aims to investigate the potential correlation between RC and HUA in American adults without receiving lipid-lowering treatment.

## Methods

### Subjects and research design

The data analyzed obtained from NHANES (2013–2020) were analyzed in this study, with a stratified, multi-stage probability and complex sample of the uninstituted population in the United States. The cross-sectional surveys were conducted by NCHS. Further information regarding NHANES methods can be accessed at www.cdc.gov/nchs/NHANEs/.

**Fig. 1:**
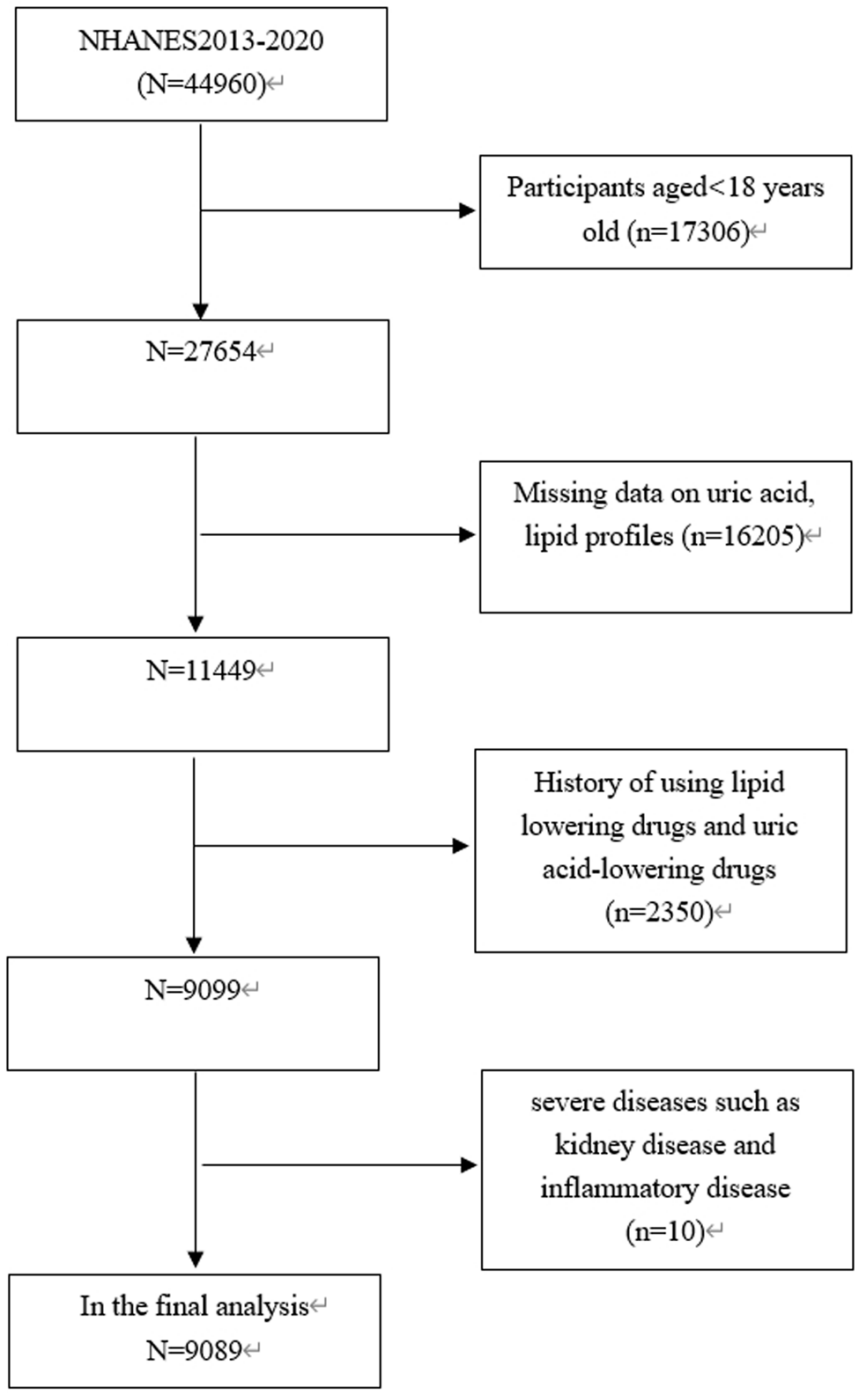
Flowchart of the sample selection from the 2013–2020 NHANES

The study focuses exclusively on subjects aged 18 years old and above (*n* = 27,654), among which, 18,565 subjects were eliminated: (1) Those with missing data on uric acid, lipid profiles; (2) Those with a history of using lipid lowering drugs and uric acid-lowering drugs; (3) Those with severe diseases such as kidney disease and inflammatory disease. Consequently, a total of 9089 subjects aged 18–80 years old were involved in the final analysis (Fig. 1).

The implementation of NHANES was approved by NCHS Ethics Review Board, and all subjects have provided informed consent in written.

### Anthropometric measurements

The following data including history of diabetes, alcohol intake, race (non-Hispanic white, non-Hispanic black, Hispanic (Mexican-American and other Hispanic), and other race/multiracial), physical activity, education and physical measurements, including weight, WC, height and blood pressure were collected at admission.

TC, glycosylated hemoglobin (HbA1c), LDL-C, uric acid (UA), fasting plasma glucose (FPG), TG, alanine aminotransferase (ALT), creatinine, glutamyl transpeptidase (GGT), aspartate aminotransferase (AST), albumin and HDL-C were collected in blood samples. Less than 3% of values were missed in total. Multiple imputation was performed for missing values. The detailed measuring method and acquisition process of each variable are available at www.cdc.gov/nchs/nhanes.

### Definition of lipid‑related indicators

Serum or plasma TC, HDL-C, and LDL-C were measured in participants who fasted for more than 8.5 h while less than 24 h in the NHANES laboratory. Specifically, TC was measured with the enzymatic assay and HDL-C was measured with the immunoassay. LDL-C was calculated from the measured values of total cholesterol (TC), triglycerides, and HDL-C according to the Friedewald calculation: [LDL-C] = [TC]—[HDL-C]—[TG/5]. The concentration of triglycerides in serum or plasma was determined with the timed-endpoint method. The 2019 European Society of Cardiology and European Atherosclerosis Society (ESC/EAS) guidelines for the management of dyslipidaemias recommend the use of TC minus HDL-C and LDL-C for RC calculation [23].

HUA was defined as a uric acid level exceeding 7 mg/dL in males and 6 mg/dL in females [24]. WC of central obesity was defined as ≥ 102 cm in males and ≥ 88 cm in females [25]. Lean weight was defined as BMI < 18.5 kg/m^2^, normal weight was defined as 18.5 ≤ BMI < 25 kg/m^2^, overweight defined as 25 ≤ BMI < 30 kg/m^2^, obesity defined as BMI ≥ 30 kg/m^2^. Waist-to-height ratio (WHtR) = WC (cm)/height (cm).

### Statistical analysis

In this study, continuous data were presented as weighted mean ± SD, and categorical variables were represented as weighted proportions. The participants were stratified into four quartiles according to the levels of RC (Q1: <13.14; Q2: 13.14–20.10; Q3: 20.10-29.96; Q4: >29.96 in male group, Q1: <12.06; Q2: 12.06–17.94; Q3: 17.94–28.89; Q4: >28.89 in female group). To assess disparities among the quartiles, the categorical variables were determined with weighted χ2 test. The correlation between RC and the presence of HUA was assessed through Binary logistic regression models. In Model 1, no covariate was adjusted. In Model 2, the covariates that change at least 10% of the initial regression coefficient of the matching risk of RC-related HUA was regarded as confounding factors and adjusts them (race, age and WHtR were adjusted). In Model 3, it was considered to be a fully adjustment since all non-collinear variables were adjusted (WHtR, race, age, moderate activities, diabetes, education level, drinking, waist circumference, DBP, serum creatinine, TG, ALT, SBP, AST, GGT, FPG, albumin were adjusted). Subgroup analyses on key variables (gender, age, race, BMI, WC, diabetes, moderate activities as well as drinking) were also conducted to assess the effect stratified by prespecified risk factors and the potential interaction effect. The potential nonlinear correlation between RC and HUA probabilities as investigated through a smooth curve fitting. Statistical analysis was performed with EmpowerStats software and R, with the significance, *P* < 0.05.

## Results

### Baseline characteristics

A total of 9089 participants aged from 18 to 80 were included in this study, with the prevalence of HUA of 16.9%. As shown in Table 1, the prevalence of HUA reached 13.9% in females and 20.2% in males, respectively. Age, proportion individuals with diabetes, WC, BMI, WHtR, SBP, DBP, ALT, AST, GGT, TC, TG, uric acid LDL-C and RC levels were all higher in HUA patients than those in non-HUA patients with both genders (*P* < 0.001).

**Table 1:** Baseline characteristics of the study population stratified by HUA and gender

	Male		*P*-value	Female		*P*-value
	HUA	Non-HUA		HUA	Non-HUA	
Number	860	3404		673	4152	
Age, year	47.1 ± 18.4	44.5 ± 17.6	< 0.001	52.1 ± 18.1	43.7 ± 16.8	
Race, %			0.013			< 0.001
Mexican American	13.3	15.7		7.4	15.9	
Other Hispanic	9.0	10.1		9.8	11.6	
Non-Hispanic White	33.7	36.0		37.6	32.5	
Non-Hispanic Black	25.9	21.0		28.7	22.3	
Other Race	18.1	17.2		16.5	17.7	
Moderate activities, %			0.561			0.038
Yes	42.0	40.9		37.7	42.9	
No	58.0	59.1		62.3	57.1	
Diabetes			0.021			< 0.001
Yes	11.3	8.5		11.4	6.8	
No	88.7	81.5		88.6	93.2	
Education level			0.001			0.551
Less than high school	17.7	23.0		17.9	18.9	
High school or above	82.3	77.0		82.1	81.1	
drinking, %			0.237			0.168
Current or ever	51.9	49.6		47.5	44.7	
Never	48.1	50.4		52.5	55.3	
Height, cm	174.7 ± 7.6	174.1 ± 7.6	0.042	160.3 ± 7.4	160.7 ± 6.9	0.186
Weight, cm	97.2 ± 26.4	83.6 ± 19.1	< 0.001	91.4 ± 28.0	74.8 ± 21.1	< 0.001
Body mass index, Kg/m2	31.8 ± 8.1	27.5 ± 5.6	< 0.001	35.3 ± 9.8	28.9 ± 7.6	< 0.001
Waist circumference, cm	107.4 ± 17.5	97.0 ± 15.3	< 0.001	109.8 ± 19.4	95.3 ± 17.0	< 0.001
WhtR	0.61 ± 0.10	0.56 ± 0.09	< 0.001	0.68 ± 0.12	0.59 ± 0.10	< 0.001
Systolic blood pressure, mmHg	127.0 ± 17.5	124.1 ± 17.2	0.001	127.0 ± 20.7	118.7 ± 17.8	< 0.001
Diastolic blood pressure, mmHg	73.5 ± 12.3	71.1 ± 12.4	< 0.001	70.4 ± 13.4	68.4 ± 11.7	0.002
Hemoglobin A1c, %	5.7 ± 0.9	5.7 ± 1.1	0.778	5.8 ± 0.8	5.6 ± 1.0	< 0.001
FPG, mg/dL	109.4 ± 25.0	109.4 ± 34.9	0.997	110.0 ± 25.7	103.9 ± 31.2	< 0.001
ALT, U/L	31.4 ± 22.2	26.7 ± 21.0	< 0.001	21.7 ± 12.7	18.7 ± 16.6	< 0.001
AST, U/L	27.7 ± 16.5	25.3 ± 22.5	0.003	22.7 ± 10.6	20.9 ± 18.4	0.013
GGT, U/L	41.0 ± 46.9	32.0 ± 53.5	< 0.001	31.2 ± 37.5	22.7 ± 31.6	< 0.001
Albumin, mg/dL	4219 ± 380	4253 ± 346	0.017	4017 ± 3420	4031 ± 355	0.343
Creatinine, mg/dL	1.08 ± 0.38	0.95 ± 0.22	< 0.001	75.2 ± 26.0	63.7 ± 18.8	< 0.001
Uric acid, mg/dL	7.97 ± 0.86	5.57 ± 0.89	< 0.001	6.93 ± 0.92	4.43 ± 0.85	< 0.001
Total cholesterol, mg/dL	190.4 ± 40.9	186.7 ± 39.9	0.015	198.1 ± 43.5	190.6 ± 41.2	< 0.001
Triglycerides, mg/dL	145.6 ± 78.7	117.4 ± 68.5	< 0.001	139.2 ± 72.9	103.4 ± 57.1	< 0.001
HDL-cholesterol, mg/dL	46.8 ± 12.9	50.5 ± 13.9	< 0.001	53.9 ± 15.6	59.5 ± 16.6	< 0.001
LDL-cholesterol, mg/dL	116.2 ± 35.2	113.6 ± 35.1	0.050	118.3 ± 38.5	111.1 ± 34.8	< 0.001
RC, mg/dL	27.4 ± 15.6	22.7 ± 13.9	< 0.001	26.0 ± 14.8	20.0 ± 12.0	< 0.001

Values are mean±SD or number (%). *P* < 0.05 was deemed significant. BMI, body mass index; FPG, fasting blood glucose; HbA1c, glycosylated hemoglobin; TC, total cholesterol; TG, triglyceride; HDL-c, High density lipoprotein cholesterol; LDL-c, Low density lipoprotein cholesterol; GGT, glutamyl transpeptidase

### Correlation between RC and metabolic parameters

The correlation between RC and metabolic parameters, as measured by Spearman’s correlation coefficient, can be found in Table 2. It was evident that RC was positively correlated with BMI, WC, WHtR, SBP, DBP, FPG, TC, TG, LDL-C, uric acid, and negatively correlated with HDL-C (Table 2).

**Table 2:** Spearmen’s correlation of RC levels with clinical and biochemical parameters

Variable	Male		Female	
	r	P	r	P
BMI	0.240	< 0.001	0.217	< 0.001
WC	0.265	< 0.001	0.280	< 0.001
WhtR	0.295	< 0.001	0.302	< 0.001
SBP	0.086	< 0.001	0.197	< 0.001
DBP	0.127	< 0.001	0.111	< 0.001
FPG	0.186	< 0.001	0.256	< 0.001
TC	0.400	< 0.001	0.421	< 0.001
TG	0.900	< 0.001	0.867	< 0.001
HDL-C	-0.436	< 0.001	-0.358	< 0.001
LDL-C	0.246	< 0.001	0.318	< 0.001
Uric acid	0.208	< 0.001	0.215	< 0.001

### Linear correlation between RC and HUA

**Fig. 2:**
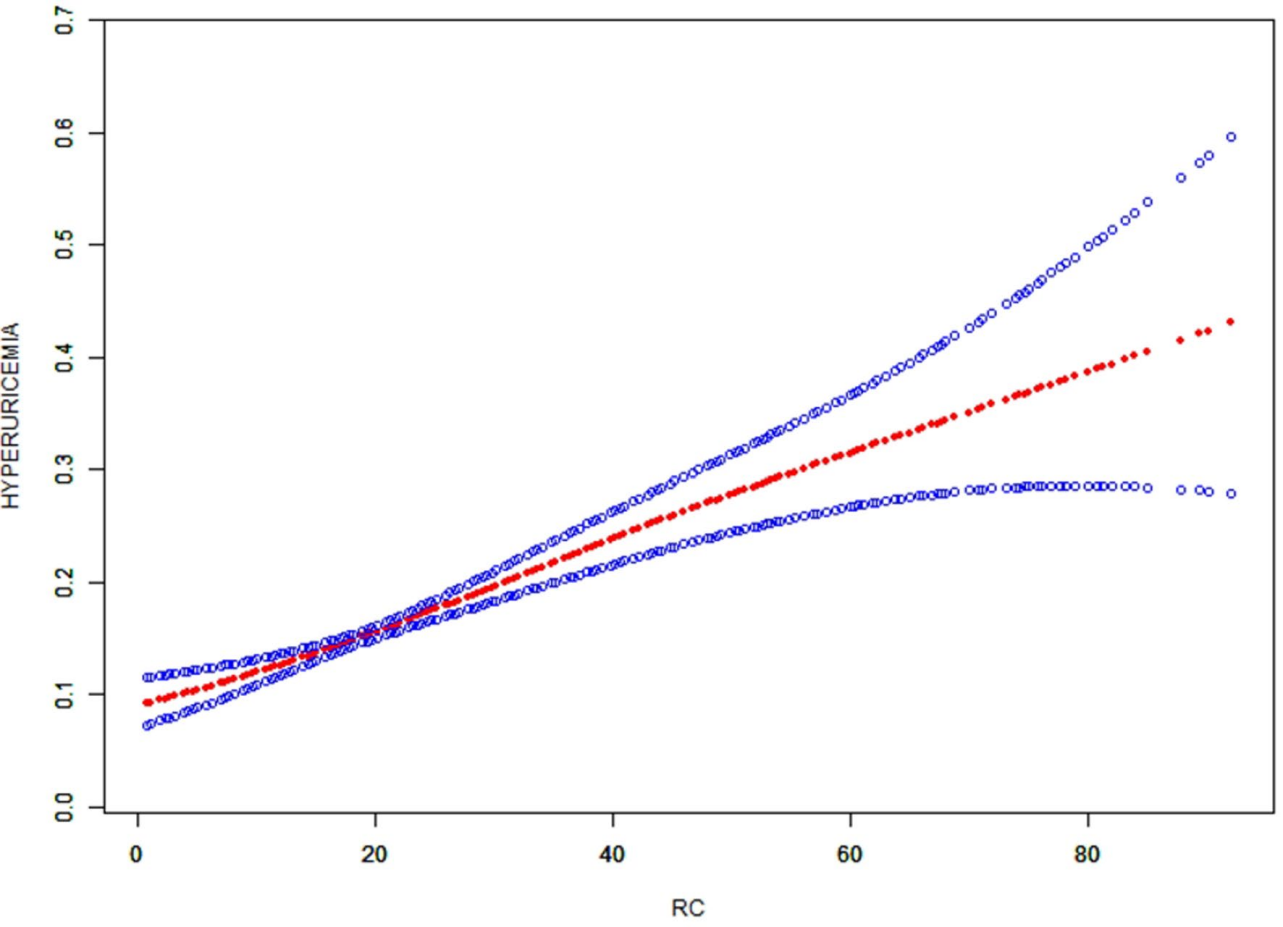
The smooth curve fit for the association between RC and hyperuricemia

Three weighted multivariate regression models were developed to examine the correlation between HUA and RC (Table 3). In the unadjusted model, RC was positively correlated with HUA probabilities (OR = 1.021 in male and 1.032 in female). The correlation still existed in Model 2(OR = 1.022 in male and 1.030 in female) and Model 3(OR = 1.022 in male and 1.031 in female). Moreover, compared with the lowest level of RC (Q1) in Model 3 (*P* < 0.001), HUA of the subjects in quartiles 3 and 4 increased. To further investigate the correlation between RC and HUA, a generalized additive model and smooth curve fittings were adopted (Fig. 2). Among all participants, RC had a linear correlation with HUA without threshold or saturation effects.

**Table 3:** Association between RC and hyperuricemia by logistic regression analysis

	Model1 OR (95% CI) *P* value	Model2 OR (95% CI) *P* value	Model3 OR (95% CI) *P* value
Male			
RC continuous	1.021 (1.016, 1.026), < 0.001	1.022 (1.017, 1.027), < 0.001	1.022 (1.015, 1.029), < 0.001
RC(Quartile)			
Q1	Reference	Reference	Reference
Q2	1.47 (1.15, 1.85), 0.002	1.31 (1.02, 1.68), 0.036	1.29 (0.91, 1.82), 0.154
Q3	1.87 (1.49, 2.35), < 0.001	1.43 (1.12, 1.82), 0.005	1.54 (1.10, 2.16), 0.013
Q4	2.52 (2.02, 3.15), < 0.001	1.87 (1.47, 2.37), < 0.001	2.43 (1.75, 3.38), < 0.001
P for trend	< 0.001	< 0.001	< 0.001
Female			
RC continuous	1.032 (1.026, 1.038), < 0.001	1.030 (1.024, 1.036), < 0.001	1.031(1.023, 1.040), < 0.001
RC(Quartile)			
Q1	Reference	Reference	Reference
Q2	1.56 (1.19, 2.06), 0.001	1.41 (1.04, 1.91), 0.026	1.48 (0.98, 2.24), 0.066
Q3	1.87 (1.44, 2.44), < 0.001	1.47 (1.09, 1.98), 0.011	1.54 (1.02, 2.31), 0.039
Q4	3.39 (2.65, 4.35), < 0.001	2.51 (1.89, 3.33), < 0.001	3.37 (2.30, 4.95), < 0.001
P for trend	< 0.001	< 0.001	< 0.001

Model I: None covariates were adjusted; Model II: age, race and WhtR were adjusted; Model III: moderate activities, diabetes, education level, drinking, SBP, DBP, FPG, TG, ALT, AST, GGT, serum creatinine, albumin were adjusted

**Fig. 3:**
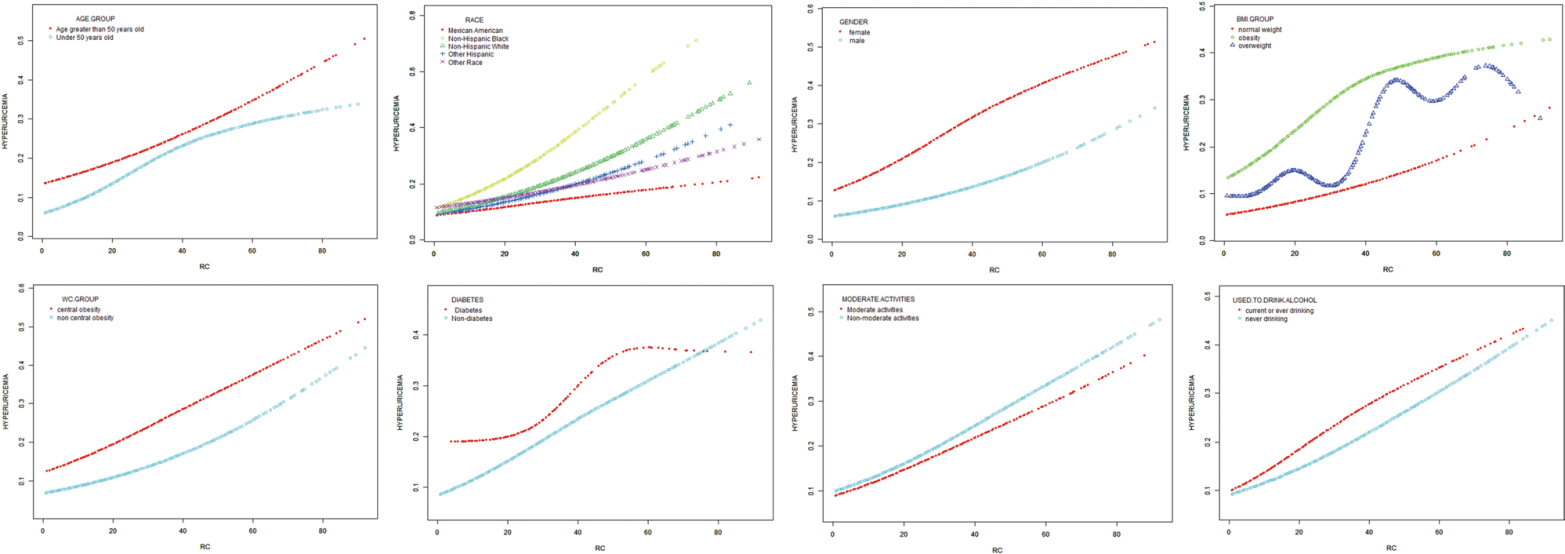
Subgroups analysis for the association between RC and hyperuricemia by gender, age, BMI, WC, race, diabetes, moderate activities and drinking

### Subgroup analysis on correlation between RC and HUA

The consistency of the correlation between RC and the odds ratio of HUA across different demographic contexts was evaluated through a comprehensive subgroup analysis. Due to a small sample size with a BMI less than 18.5 kg/m^2^ and a prevalence rate of only 4.5% for hyperuricemia, it was not included in the subgroup analysis. As shown in Table 4, the positive correlation between RC and the odds ratio of HUA was not significantly affected by race, BMI, WC, moderate activities and drinking (*P* > 0.05 for all interactions). The correlation between RC and HUA odds ratio was stronger among female participants (OR = 1.031, *P* interaction < 0.001), among participants with age < 50 years old (OR = 1.029, *P* < 0.001), among non-diabetes (OR = 1.030, *P* interaction < 0.001).

**Table 4:** Association between RC and hyperuricemia stratified by gender, age, race, BMI, WC, diabetes, moderate activities and drinking

	OR (95%CI) *p* value	*P* for interaction
Stratified by gender		0.035
Male	1.022 (1.015, 1.029), < 0.001	
Female	1.031(1.023, 1.040), < 0.001	
Stratified by race		0.085
Mexican American	1.018 (1.003, 1.033), 0.017	
Other Hispanic	1.028 (1.011, 1.045), 0.001	
Non-Hispanic White	1.032 (1.023, 1.041), < 0.001	
Non-Hispanic Black	1.039 (1.024, 1.055), < 0.001	
Other Race	1.023 (1.010, 1.036), < 0.001	
Stratified by age		0.047
Age < 50 years old	1.029 (1.020, 1.035), < 0.001	
Age ≥ 50 years old	1.023 (1.015, 1.030), < 0.001	
Stratified by BMI		0.767
Normal weight	1.025 (1.011, 1.040), 0.001	
Overweight	1.027 (1.017, 1.036), < 0.001	
Obesity	1.019 (1.011, 1.027), < 0.001	
Stratified by WC		0.672
Central obesity	1.024 (1.020, 1.026), < 0.001	
Non central obesity	1.026 (1.016, 1.036), < 0.001	
Stratified by diabetes		0.041
Non-diabetes	1.030 (1.022, 1.032), < 0.001	
Diabetes	1.023 (1.006, 1.040), 0.008	
Stratified by Moderate activities		0.765
No	1.026 (1.019, 1.032), < 0.001	
Yes	1.026 (1.18, 1.035), < 0.001	
Stratified by drinking		0.866
Current or ever drinking	1.029 (1.019, 1.040), < 0.001	
Never	1.025 (1.019, 1.031), < 0.001	

Gender, age, race, moderate activities, diabetes, education level, drinking, BMI, waist circumference (not adjusted for in the subgroup analyses), SBP, DBP, FPG, TG, ALT, AST, GGT, serum creatinine and albumin were adjusted

The generalized additive model and fitted smoothing curve are consistent with multivariate logistic regression models for the different subgroup (Fig. 3).

## Discussion

This study discovered a strong positive correlation between RC and the prevalence of HUA in American adults, even after adjusting for various confounding factors, and exhibited a linear correlation. Through subgroup analysis and interaction assessment, a stronger correlation was discovered in females, participants with age < 50 years old and those with non-diabetes. This is the first known study of the strong positive correlation between RC and the prevalence of HUA and the presence of age, gender and diabetes differences in American adults.

With the material living conditions have been improved and unhealthy lifestyles become more prevalent, the incidence rate of HUA continues to rise [5, 26–28]. Notably, the Atherosclerosis Risk in Communities Study including 9451 Americans regularly consumed high-fructose corn syrup, such as sugar-sweetened soda, reported a 34.8% development rate of HUA during a 6-year follow-up [29]. In this study, the prevalence of HUA was 16.9% among general American adults during 2013–2020. Furthermore, findings from the NHANES indicated a prevalence of HUA nearly 20% in general Americans during 2015–2017 [3]. It was evident that the prevalence of HUA in Americans keeps rising, mirroring trends observed in other countries globally.

Recent clinical and epidemiological studies have highlighted the role of dyslipidemia in the development of HUA, with NHANES III revealing a significant correlation between the levels of TG and TC and the levels of UA in serum of general adults [21]. Nakanishi et al. discovered that basal TG continued to serve as a significant predictor of new-onset HUA, even when patients with diabetes mellitus and those on long-term medications for specific chronic conditions were excluded [30]. A retrospective study of 3884 participants from medical examinations in Gansu, China, revealed a positive correlation between elevated TG and HUA [20]. RC is the cholesterol found in Triglyceride-Rich Lipoproteins (TRLs), encompassing very low-density lipoproteins, intermediate-density lipoproteins, and chylomicron remnants [31]. Cholesterol in TRLs has a more direct impact on the risk of cardiovascular disease [32]. A multi-center cohort study, with an 18-year follow-up period, demonstrated that elevated levels of RC pose a risk for the onset of atherosclerotic cardiovascular disease (ASCVD), beyond traditional cardiovascular risk factors such as LDL-C and ApoB [33]. Some scholars suggest that RC, rather than LDL-C, is linked to the development of cardiovascular disease [33]. Further studies have confirmed that RC not only predicts the risk for cardiovascular disease, but also the risk of metabolic disease [34–36]. HUA and cardiovascular diseases share common risk factors [37]. This cross-sectional study also confirmed that RC is positively correlated with the HUA.

This study showed that RC was positively correlated with DBP, BMI, WC, TG, FPG, SBP, TC, LDL-C and negatively correlated with HDL-C, which is consistent with previous studies. Additionally, it was found that the correlation between RC and TG was the strongest compared to that between RC and that with other components of MetS. This is consistent with previous studies, suggesting that TG is primarily transported by remnants and that the concentration of TG significantly increases with elevated levels of RC [13, 38]. These findings collectively suggest a strong correlation between RC levels and metabolic disorders.

Furthermore, whether the correlation between RC and HUA was influenced by various established risk factors was investigated through stratified analyses. This study revealed notable gender disparities in the correlation between RC and HUA, with a notably stronger correlation observed in females than that in males. Interestingly, a similar trend has been observed in the correlation between RC and the risks of chronic kidney disease, diabetes, and NAFLD [39–41]. Although the exact mechanism underlying these gender-specific differences are still uncertain, gender hormones such as estrogen may play a role. Existing literature supports the influential role of estrogen signaling via Estrogen Receptor alpha (ERα) in modulating lipid and glucose metabolism [42]. Therefore, the decreased in estrogen levels following menopause may result in the dysregulation of lipid metabolism, leading the increased the susceptibility of females to developing HUA.

In those at the age < 50 years old or those without diabetes, RC was associated with higher odds ratio for HUA than that in those older than 50 years or those with diabetes. Diabetic patients generally have lower levels of SUA than those without diabetes [43–45]. Decreased preglomerular resistance in diabetic patients helps to increase glomerular hyperfiltration, further promotes the excretion of UA, and leads to hypouricemia [46, 47]. Similarly, the islet function of diabetic patients was often damaged, which led to a decrease in insulin secretion in the body, downregulating the expression of renal urate transporters, reducing the reabsorption of UA, and reducing SUA levels [48]. Additionally, dietary irregularities and insufficient exercise in young individuals can lead to excessive fat accumulation, potentially influencing RC [49]. Therefore, HUA usually has a stronger correlation with RC in the younger or without diabetes population.

Several plausible mechanisms can be postulated to elucidate the correlation between RC and the development of HUA. First of all, the elevation of RC levels in body will lead to an induction of heightened production and utilization of free fatty acids, consequently accelerating the catabolism of adenosine triphosphate and resulting in an augmented production of serum uric acid [50]. Secondly, an elevated RC level has been found to be independently associated with a reduced estimated glomerular filtration rate and an increased risk of renal impairment, potentially leading to a diminished excretion of uric acid [41]. Lastly, RC can serve as a surrogate marker for IR [51], and IR is a factor closely related to the pathogenesis of HUA. IR has been shown that it can enhance renal urate reabsorption through the stimulation of URAT1 [52] and/or Nadependent anion co-transporter in brush border membranes of renal proximal tubule [52, 53]. This finding strengthened on previous studies showed a pathogenesis among hyperuricemia and dyslipidemia [54, 55].

### Study strengths and limitations

Notably, the study benefits from a large sample size and the national representativeness of Americans. In addition, various indexes in the model were adjusted to enhance the reliability of the findings. Nonetheless, this study is subject to certain limitations. Firstly, the establishment of a causal correlation between RC and HUA was not feasible through cross-sectional studies. Secondly, the measurement of RC is not currently a standard component of clinical blood lipid testing through direct means, thus only RC levels can be calculated. Thirdly, American adults are restricted to the study, necessitating further prospective cohort research to validate and generalize the present results in a broader population.

## Conclusion

In summary, elevated RC was independently associated with HUA in a sizable cohort of American adults. This correlation was particularly pronounced among females, those under 50 years old, and those without diabetes. The RC could serve as an effective biomarker for assessing the risk of HUA.

## Data Availability

The datasets generated and analysis during the current study are available in the NHANES, www.cdc.gov/nchs/NHANEs/.

## References

[1] Smith E, Hoy D, Cross M, Merriman TR, Vos T, Buchbinder R, Woolf A, March L (2014). The global burden of gout: estimates from the global burden of Disease 2010 study. Ann Rheum Dis.

[2] George C, Leslie SW, Minter DA. Hyperuricemia. In *StatPearls* Treasure Island (FL) ineligible companies. Disclosure: Stephen Leslie declares no relevant financial relationships with ineligible companies. Disclosure: David Minter declares no relevant financial relationships with ineligible companies.; 2024.

[3] Borghi C, Agabiti-Rosei E, Johnson RJ, Kielstein JT, Lurbe E, Mancia G, Redon J, Stack AG, Tsioufis KP (2020). Hyperuricaemia and gout in cardiovascular, metabolic and kidney disease. Eur J Intern Med.

[4] Fang XY, Qi LW, Chen HF, Gao P, Zhang Q, Leng RX, Fan YG, Li BZ, Pan HF, Ye DQ (2022). The Interaction between Dietary Fructose and Gut Microbiota in Hyperuricemia and gout. Front Nutr.

[5] Chen-Xu M, Yokose C, Rai SK, Pillinger MH, Choi HK (2019). Contemporary prevalence of gout and Hyperuricemia in the United States and Decadal trends: the National Health and Nutrition Examination Survey, 2007–2016. Arthritis Rheumatol.

[6] Xia Y, Wu Q, Wang H, Zhang S, Jiang Y, Gong T, Xu X, Chang Q, Niu K, Zhao Y (2020). Global, regional and national burden of gout, 1990–2017: a systematic analysis of the global burden of Disease Study. Rheumatology (Oxford).

[7] Nordestgaard BG, Varbo A (2014). Triglycerides and cardiovascular disease. Lancet.

[8] Basu D, Bornfeldt KE (2020). Hypertriglyceridemia and atherosclerosis: using Human Research to Guide mechanistic studies in animal models. Front Endocrinol (Lausanne).

[9] Varbo A, Benn M, Tybjaerg-Hansen A, Nordestgaard BG (2013). Elevated remnant cholesterol causes both low-grade inflammation and ischemic heart disease, whereas elevated low-density lipoprotein cholesterol causes ischemic heart disease without inflammation. Circulation.

[10] Nordestgaard BG (2016). Triglyceride-Rich lipoproteins and Atherosclerotic Cardiovascular Disease: New insights from Epidemiology, Genetics, and Biology. Circ Res.

[11] Ye X, Kong W, Zafar MI, Chen LL (2019). Serum triglycerides as a risk factor for cardiovascular diseases in type 2 diabetes mellitus: a systematic review and meta-analysis of prospective studies. Cardiovasc Diabetol.

[12] Nordestgaard BG, Tybjaerg-Hansen A (2011). Genetic determinants of LDL, lipoprotein(a), triglyceride-rich lipoproteins and HDL: concordance and discordance with cardiovascular disease risk. Curr Opin Lipidol.

[13] Varbo A, Benn M, Nordestgaard BG (2014). Remnant cholesterol as a cause of ischemic heart disease: evidence, definition, measurement, atherogenicity, high risk patients, and present and future treatment. Pharmacol Ther.

[14] de Graaf J, van der Vleuten GM, ter Avest E, Dallinga-Thie GM, Stalenhoef AF (2007). High plasma level of remnant-like particles cholesterol in familial combined hyperlipidemia. J Clin Endocrinol Metab.

[15] Lewis GF, Carpentier A, Adeli K, Giacca A (2002). Disordered fat storage and mobilization in the pathogenesis of insulin resistance and type 2 diabetes. Endocr Rev.

[16] Arner P, Bernard S, Salehpour M, Possnert G, Liebl J, Steier P, Buchholz BA, Eriksson M, Arner E, Hauner H (2011). Dynamics of human adipose lipid turnover in health and metabolic disease. Nature.

[17] Xiao C, Hsieh J, Adeli K, Lewis GF (2011). Gut-liver interaction in triglyceride-rich lipoprotein metabolism. Am J Physiol Endocrinol Metab.

[18] Jin J, Meng X, Wang D, Han B, Wu T, Xie J, Zhang Q, Xie D, Zhang Z (2023). Association between ambient temperature and cardiovascular diseases related hospital admissions in Lanzhou, China. Heliyon.

[19] Rafiullah M, Siddiqui K, Al-Rubeaan K (2020). Association between serum uric acid levels and metabolic markers in patients with type 2 diabetes from a community with high diabetes prevalence. Int J Clin Pract.

[20] Hou YL, Yang XL, Wang CX, Zhi LX, Yang MJ, You CG (2019). Hypertriglyceridemia and hyperuricemia: a retrospective study of urban residents. Lipids Health Dis.

[21] Peng TC, Wang CC, Kao TW, Chan JY, Yang YH, Chang YW, Chen WL. Relationship between hyperuricemia and lipid profiles in US adults. *Biomed Res Int* 2015, 2015:127596. 10.1155/2015/127596 PMC4299312 25629033

[22] Zhang X, Meng Q, Feng J, Liao H, Shi R, Shi D, Renqian L, Langtai Z, Diao Y, Chen X (2018). The prevalence of hyperuricemia and its correlates in Ganzi Tibetan Autonomous Prefecture, Sichuan Province, China. Lipids Health Dis.

[23] Mach F, Baigent C, Catapano AL, Koskinas KC, Casula M, Badimon L, Chapman MJ, De Backer GG, Delgado V, Ference BA (2020). 2019 ESC/EAS guidelines for the management of dyslipidaemias: lipid modification to reduce cardiovascular risk. Eur Heart J.

[24] Liu R, Han C, Wu D, Xia X, Gu J, Guan H, Shan Z, Teng W. Prevalence of Hyperuricemia and Gout in Mainland China from 2000 to 2014: A Systematic Review and Meta-Analysis. *Biomed Res Int* 2015, 2015:762820. 10.1155/2015/762820 PMC4657091 26640795

[25] Moura L, Pagotto V, Camargo Pereira C, de Oliveira C, Silveira EA. Does abdominal obesity increase All-Cause, Cardiovascular Disease, and Cancer Mortality risks in older adults? A 10-Year Follow-Up analysis. Nutrients 2022, 14. 10.3390/nu14204315 PMC9607321 36296999

[26] Bardin T, Richette P (2017). Impact of comorbidities on gout and hyperuricaemia: an update on prevalence and treatment options. BMC Med.

[27] Kumar AUA, Browne LD, Li X, Adeeb F, Perez-Ruiz F, Fraser AD, Stack AG (2018). Temporal trends in hyperuricaemia in the Irish health system from 2006–2014: a cohort study. PLoS ONE.

[28] Wang H, Zhang H, Sun L, Guo W (2018). Roles of hyperuricemia in metabolic syndrome and cardiac-kidney-vascular system diseases. Am J Transl Res.

[29] Bomback AS, Derebail VK, Shoham DA, Anderson CA, Steffen LM, Rosamond WD, Kshirsagar AV (2010). Sugar-sweetened soda consumption, hyperuricemia, and kidney disease. Kidney Int.

[30] Nakanishi N, Yoshida H, Nakamura K, Suzuki K, Tatara K (2001). Predictors for development of hyperuricemia: an 8-year longitudinal study in middle-aged Japanese men. Metabolism.

[31] Jorgensen AB, Frikke-Schmidt R, West AS, Grande P, Nordestgaard BG, Tybjaerg-Hansen A (2013). Genetically elevated non-fasting triglycerides and calculated remnant cholesterol as causal risk factors for myocardial infarction. Eur Heart J.

[32] Sandesara PB, Virani SS, Fazio S, Shapiro MD (2019). The forgotten lipids: triglycerides, remnant cholesterol, and atherosclerotic Cardiovascular Disease Risk. Endocr Rev.

[33] Quispe R, Martin SS, Michos ED, Lamba I, Blumenthal RS, Saeed A, Lima J, Puri R, Nomura S, Tsai M (2021). Remnant cholesterol predicts cardiovascular disease beyond LDL and ApoB: a primary prevention study. Eur Heart J.

[34] Chen J, Su Y, Su X, Luo F (2023). Remnant cholesterol has a non-linear association with non-alcoholic fatty liver disease. Diabetes Res Clin Pract.

[35] Huang H, Xie J, Zeng Y, Liu Z, Miao M, Xu L, Xu C (2023). Remnant cholesterol independently predicts the development of nonalcoholic fatty liver disease. J Clin Endocrinol Metab.

[36] Szili-Torok T, Sokooti S, Oste MCJ, Gomes-Neto AW, Dullaart RPF, Bakker SJL, Tietge UJF (2022). Remnant lipoprotein cholesterol is associated with incident new onset diabetes after transplantation (NODAT) in renal transplant recipients: results of the TransplantLines Biobank and cohort studies. Cardiovasc Diabetol.

[37] Pillinger MH, Bangalore S, Klein AB, Baumgartner S, Morlock R (2017). Cardiovascular Disease and gout: real-world experience evaluating patient characteristics, Treatment Patterns, and Health Care utilization. J Manag Care Spec Pharm.

[38] Varbo A, Benn M, Tybjaerg-Hansen A, Jorgensen AB, Frikke-Schmidt R, Nordestgaard BG (2013). Remnant cholesterol as a causal risk factor for ischemic heart disease. J Am Coll Cardiol.

[39] Zou Y, Lan J, Zhong Y, Yang S, Zhang H, Xie G (2021). Association of remnant cholesterol with nonalcoholic fatty liver disease: a general population-based study. Lipids Health Dis.

[40] Hu X, Liu Q, Guo X, Wang W, Yu B, Liang B, Zhou Y, Dong H, Lin J (2022). The role of remnant cholesterol beyond low-density lipoprotein cholesterol in diabetes mellitus. Cardiovasc Diabetol.

[41] Yan P, Xu Y, Miao Y, Bai X, Wu Y, Tang Q, Zhang Z, Yang J, Wan Q (2021). Association of remnant cholesterol with chronic kidney disease in middle-aged and elderly Chinese: a population-based study. Acta Diabetol.

[42] Palmisano BT, Zhu L, Stafford JM (2017). Role of Estrogens in the regulation of liver lipid metabolism. Adv Exp Med Biol.

[43] Okada M, Ueda K, Omae T, Takeshita M, Hirota Y (1982). The relationship of serum uric acid to hypertension and ischemic heart disease in Hisayama population, Japan. J Chronic Dis.

[44] Tuomilehto J, Zimmet P, Wolf E, Taylor R, Ram P, King H (1988). Plasma uric acid level and its association with diabetes mellitus and some biologic parameters in a biracial population of Fiji. Am J Epidemiol.

[45] Li H, Sun M, Huang C, Wang J, Huang Y. Association between Glycosylated Hemoglobin and Serum Uric Acid: A US NHANES 2011–2020. *Int J Endocrinol* 2024, 2024:5341646. 10.1155/2024/5341646 PMC10957249 38515506

[46] Cortinovis M, Perico N, Ruggenenti P, Remuzzi A, Remuzzi G (2022). Glomerular hyperfiltration. Nat Rev Nephrol.

[47] Golik A, Weissgarten J, Cotariu D, Cohen N, Zaidenstein R, Ramot Y, Averbukh Z, Modai D (1993). Renal uric acid handling in non-insulin-dependent diabetic patients with elevated glomerular filtration rates. Clin Sci (Lond).

[48] Toyoki D, Shibata S, Kuribayashi-Okuma E, Xu N, Ishizawa K, Hosoyamada M, Uchida S (2017). Insulin stimulates uric acid reabsorption via regulating urate transporter 1 and ATP-binding cassette subfamily G member 2. Am J Physiol Ren Physiol.

[49] Correa-Rodriguez M, Gonzalez-Jimenez E, Fernandez-Aparicio A, Luis Gomez-Urquiza J, Schmidt-RioValle J, Rueda-Medina B (2021). Dietary Energy Density is Associated with Body Mass Index and Fat Mass in Early Adulthood. Clin Nurs Res.

[50] Balasubramanian T (2003). Uric acid or 1-methyl uric acid in the urinary bladder increases serum glucose, insulin, true triglyceride, and total cholesterol levels in Wistar rats. ScientificWorldJournal.

[51] Fukushima M, Taniguchi A, Nakai Y, Sakai M, Doi K, Nin K, Oguma T, Nagasaka S, Tokuyama K, Seino Y (2001). Remnant-like particle cholesterol and insulin resistance in nonobese nonhypertensive Japanese glucose-tolerant relatives of type 2 diabetic patients. Diabetes Care.

[52] Enomoto A, Kimura H, Chairoungdua A, Shigeta Y, Jutabha P, Cha SH, Hosoyamada M, Takeda M, Sekine T, Igarashi T (2002). Molecular identification of a renal urate anion exchanger that regulates blood urate levels. Nature.

[53] Perez-Ruiz F, Aniel-Quiroga MA, Herrero-Beites AM, Chinchilla SP, Erauskin GG, Merriman T (2015). Renal clearance of uric acid is linked to insulin resistance and lower excretion of sodium in gout patients. Rheumatol Int.

[54] Lu W, Song K, Wang Y, Zhang Q, Li W, Jiao H, Wang G, Huang G (2012). Relationship between serum uric acid and metabolic syndrome: an analysis by structural equation modeling. J Clin Lipidol.

[55] Conen D, Wietlisbach V, Bovet P, Shamlaye C, Riesen W, Paccaud F, Burnier M (2004). Prevalence of hyperuricemia and relation of serum uric acid with cardiovascular risk factors in a developing country. BMC Public Health.

